# Rapid Method for Estimating Polyhydroxybutyrate Accumulation in Bacteria Using Sodium Hypochlorite

**DOI:** 10.21769/BioProtoc.5130

**Published:** 2024-12-05

**Authors:** Ingrid E. Redersdorff, Ailen N. Rodríguez, Mariana Escobar, Claudia A. Studdert, M. Karina Herrera Seitz

**Affiliations:** 1 Instituto de Investigaciones Biológicas, CONICET - Universidad Nacional de Mar del Plata, Buenos Aires, Argentina; 2 Instituto de Agrobiotecnología del Litoral, CONICET - Universidad Nacional del Litoral, Santa Fe, Argentina

**Keywords:** Polyhydroxybutyrate, Sodium hypochlorite oxidation, *Halomonas* sp., *Alteromonas* sp., *Cobetia* sp.

## Abstract

This protocol outlines the use of the previously described sodium hypochlorite extraction method for estimating the accumulation of polyhydroxybutyrate (PHB) in bacteria. Sodium hypochlorite (NaClO) is widely used for PHB extraction as it oxidizes most components of the cells except PHB. We assessed the feasibility of using NaClO extraction for the estimation of PHB accumulation in bacterial cells (expressed as a percentage w/w). This allowed us to use a simple spectrophotometric measurement of the turbidity of the PHB extracted by NaClO as a semiquantitative estimation of PHB accumulation in the marine microorganisms *Halomonas titanicae* KHS3, *Alteromonas* sp., and *Cobetia* sp. However, this fast and easy protocol could be used for any bacterial species as long as some details are considered. This estimation exhibited a good correlation with the accumulation measured as dry cell weight or even with the accumulation measured by crotonic acid and HPLC quantifications. The key advantage of this protocol is how fast it allows an estimation of PHB accumulation in *Halomonas*, *Alteromonas*, and *Cobetia* cultures (results are available in 50 min), enabling the identification of the appropriate moment to harvest cells for further extraction, polymer characterization, and accurate quantification using more reliable and time-consuming methods. This protocol is very useful during bacterial cultivation for a quick evaluation of PHA accumulation without requiring (i) large volumes of cultures, (ii) a long time for analysis compared to dry cell weight, (iii) preparation of standard curves with sulfuric acid hydrolysis for crotonic acid quantification, or (iv) specific equipment and/or technical services for HPLC quantification.

Key features

• Fast and easy method for bacterial PHB content estimation in cultures of different marine microorganisms. It can be used in other PHB-accumulating bacteria.

• Useful to explore culture conditions to achieve maximal PHB accumulation.

• Useful to follow the kinetics of both PHB accumulation and mobilization throughout culture development.

• In cultures with high (50%–70% dry cell weight) or very low (<15%) PHB accumulation, differences are visible by the naked eye before spectrophotometric measurement.

## Graphical overview


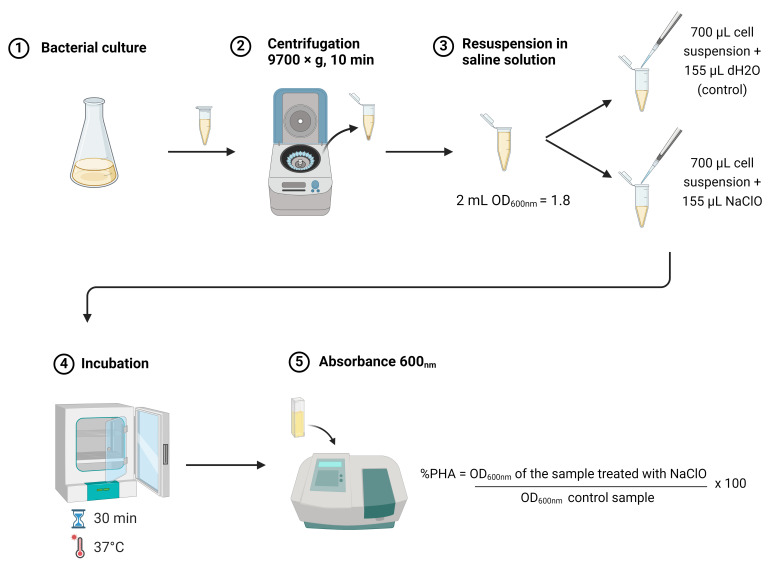


**Rapid estimation of polyhydroxybutyrate content in bacteria using modified sodium hypochlorite treatment**

## Background

Polyhydroxyalkanoates (PHAs) are bacterial-origin polymers that share several characteristics with hydrocarbon-derived plastics. Among PHAs, polyhydroxybutyrates (PHBs) are the most common type, known for their biocompatibility and biodegradability. PHAs were first described in 1927 by Lemoigne [1] in bacteria from the genus *Bacillus*, and their presence in the cell was associated with the observation of Sudan Black–stained lipid inclusions. As early as 1958, Williamson and Wilkinson [2] described a method still widely used for the extraction and purification of PHAs from cells—treatment with an alkaline solution of sodium hypochlorite (NaClO). When *Bacillus* cells were suspended in such a solution, they were almost completely dissolved, including spores if present. Williamson and Wilkinson determined the best conditions (pH, temperature, incubation time) for the hypochlorite treatment and observed that the turbidity of the cell suspension started to decrease from the beginning of the treatment and reached a constant value when the cell lysis was complete. Moreover, the residual turbidity was proportionally higher in cell suspensions containing larger amounts of lipid inclusions as judged by microscopic observation, and they were able to show that the residual turbidity was strongly correlated with the concentration of PHA (µg/mL) remaining after the treatment. Hypochlorite treatment of *Halomonas titanicae* KHS3 cells that accumulated PHAs, followed by two washes with distilled water and one wash with ethanol, rendered a material that, upon NMR analysis, was found to be pure polyhydroxybutyrate [3]. PHB content could then be quantified by measuring the dry weight of hypochlorite-resistant material relative to the total dry biomass or, more precisely, through complete acid hydrolysis of the PHB material for spectrophotometric determination. However, neither of those possibilities allowed a rapid and continuous evaluation of the onset of the accumulation stage or the moment at which the cells reach a certain level of PHB content. It was then considered that the old turbidimetric measurements could be used to standardize a rapid, simple, and inexpensive method for the estimation of PHB content in growing cultures of PHB-accumulating strains. In the study conducted by Williamson and Wilkinson in 1958 [2], it was found that high cell density suspensions resulted in excessive residual turbidity, possibly due to incomplete cell lysis. As part of our protocol, we set a maximum cell density of OD_600nm_ = 2 for the assay. By using a simple spectrophotometric measurement of the remaining turbidity after NaClO hydrolysis, our fast protocol resulted in a semiquantitative estimation of the percentage of PHB accumulation expressed as weight/weight in the marine microorganisms *Halomonas titanicae* KHS3, *Alteromonas* sp., and *Cobetia* sp. This estimation exhibited a reliable estimation of the accumulation, providing values that correlated to PHB weight/dry cell weight or even with the accumulation measured by crotonic acid and HPLC quantifications. Moreover, when propionic acid was added to the culture media, *Halomonas titanicae* KHS3 accumulated the co-polymer poly-hydroxy-butyrate-valerate (PHBV), and it was also possible to estimate PHBV accumulation using the described protocol. Therefore, although not tested here, it is highly probable that this protocol will also work for other PHAs and bacterial species.

## Materials and reagents

**Biological materials**

1. Environmental strains isolated from seawater and identified as *Halomonas titanicae* KHS3, *Alteromonas* sp., and *Cobetia* sp.

**Reagents**

1. Dipotassium phosphate (K_2_HPO_4_) (Merck, catalog number: 105104)
2. Monopotassium phosphate (KH_2_PO_4_) (Fluka, catalog number: 104873)
3. Ammonium sulfate [(NH_4_)_2_SO_4_] (Merck, catalog number: 101217)
4. Sodium chloride (NaCl) (J.T. Baker, catalog number: 3624-19)
5. Magnesium sulphate heptahydrate (MgSO_4_·7H_2_O) (Anedra, catalog number: 10034-99-8)
6. Ferric chloride hexahydrate (FeCl_3_·6H_2_O) (Fluka, catalog number: 44944)
7. Sodium hypochlorite (NaClO) [5.5% (w/v) commercially available household bleach] *
8. Yeast extract (YE) (Oxoid, catalog number: LP0021)
9. Glycerol (Gly) (BioPack, catalog number: 1620.08)
10. Distilled water
11. Bacteriological agar (Oxoid, catalog number: LP0011)
*Note: Store at room temperature. Undiluted household bleach has a shelf life of six months to one year; afterward, it degrades and loses oxidative activity.

**Solutions**

1. H1 minimal medium (see Recipes)
2. Yeast extract 10% (YE) (see Recipes)
3. Glycerol 75% (Gly) (see Recipes)
4. Saline solution (NaCl 2%) (see Recipes)

**Recipes**

*Note: All solutions must be autoclaved before use.*

1. **H1 minimal medium (200 mL)**
  **Table:** 

Reagent	Final concentration	Quantity or Volume
K_2_HPO_4_	11.2 g/L	2.24 g
KH_2_PO_4_	4.8 g/L	0.96 g
(NH_4_)_2_SO_4 _	2 g/L	0.4 g
NaCl	20 g/L	4 g
MgSO_4_·7H_2_O (1 M)	1 mM	1 mL*
FeCl_3_·6H_2_O (0.5% (w/v))	1.85 µM	0.1 mL*
H_2_O	n/a	200 mL
Total	n/a	200 mL
*Add after autoclaving.
*Note: Different carbon sources are used depending on the desired culture condition: for PHB accumulation condition, 3% glycerol; for non-PHB accumulation condition, 0.25% yeast extract. To prepare plates, add 1.5% bacteriological agar to the medium. This protocol utilizes marine halotolerant bacteria, which are cultivated in H1 minimal medium. If non-halotolerant bacteria are used, appropriate media must be prepared.*
2. **Yeast extract (YE) 10% (w/v) (20 mL)**
  **Table:** 

Reagent	Final concentration	Quantity or Volume
Yeast extract	100 g/L	2 g
H_2_O	n/a	20 mL
Total	n/a	20 mL
3. **Glycerol (Gly) 75% (v/v) (100 mL)**
  **Table:** 

Reagent	Final concentration	Quantity or Volume
Glycerol	945 g/L	75 mL
H_2_O	n/a	25 mL
Total	n/a	100 mL
4. **Saline solution (NaCl 2%) (100 mL)**
  **Table:** 

Reagent	Final concentration	Quantity or Volume
NaCl	20 g/L	2 g
H_2_O	n/a	100 mL
Total	n/a	100 mL
*Note: For the cell suspension solution when using halophilic or halotolerant microorganisms. Otherwise, use a solution with the appropriate composition (saline/buffer solution similar to the growth medium).*

**Laboratory supplies**

1. Microcentrifuge tube 2 mL (Henso Medical Co. Ltd., catalog number: HSN1411)
2. Micropipette tips 200 μL (Henso Medical Co. Ltd., catalog number: HSN1402)
3. Micropipette tips 1,000 μL (Henso Medical Co. Ltd., catalog number: HSN1402)
4. Flask 250 mL (Pyrex, catalog number: 4442-250)
5. Glass Petri dishes (Pyrex, catalog number: 3160-60)
6. Glass reagent bottle 500 mL (Duran, catalog number: 218014459)

## Equipment

1. Micropipette P1000 (100–1,000 μL) (Gilson, model: P1000)
2. Micropipette P200 (20–200 μL) (Gilson, model: P200)
3. Glass alcohol burner (Everglass, catalog number: EVG1381)
4. Mini centrifuge (Eppendorf, model: MiniSpin plus)
5. Cell density meter (Biochrom, model: Ultrospec^TM^ 10 Classic)
6. Cuvettes quartz glass (Hellma, model: 6040-UV-10-531)
7. Orbital incubator shaker (Amerex Instruments, model: Gyromax 727)
8. Autoclave 13.5 L (Ficoinox, model: SL9000)

## Procedure

- **Bacterial growth under accumulation and no-accumulation (control) conditions**
  1. Spread a fresh H1 minimal medium plus 0.25% yeast extract plate of *H. titanicae* or the desired microorganism and incubate at the appropriate temperature and time. Since this protocol could be used for any bacterial strain, the selected culture media will depend on the bacterial species.
  2. Start a fresh 3 mL overnight culture inoculated from the fresh plate from step A1.
  3. Inoculate a 50 mL culture in a 250 mL flask with an initial OD_600nm_ of approximately 0.05, using either accumulation medium (H1 medium with 3% glycerol) or non-accumulation medium (H1 medium with 0.25% yeast extract).
  4. Monitor cell growth by measuring optical density (OD_600nm_) using 1 mL of sample for spectrophotometric determination. Collect samples at desired intervals to evaluate PHB accumulation.
- **Evaluation of PHB accumulation**
  1. Determine OD_600nm_ of culture to be evaluated.
  2. Harvest an appropriate volume of culture. The volume of samples needed for PHB evaluation at each growth point will depend on the OD_600nm_ values of the culture. At each growth stage, collect enough culture to obtain 2 mL of cell suspension with OD_600nm_ between 1.7 and 2.
    Example: For a culture with an OD_600nm _of 0.45, it will be necessary to harvest between 7.55 and 8.9 mL of culture to obtain 2 mL of cell suspension with a final OD_600nm_ between 1.7 and 2.0.
    (final volume desired of cell suspension) × (final desired OD_600nm_) = (culture volume needed) × (OD_600nm_ of culture)
    2 mL × 2 = X mL × 0.45
  3. Once the required volume of culture is determined, centrifuge at 9,700× *g* for 5 min to pellet the cells. Remove the supernatant by pouring off the remaining culture medium and gently withdrawing the residue with a P200 pipette.
  4. Resuspend the cell pellet in 2 mL of 2% NaCl solution. Alternatively, cells can be resuspended in 2 mL of fresh medium without a carbon source. This step should be adapted to each bacterial strain requirement. For example, non-halophilic microorganisms’ cells could be resuspended in a low ionic-strength buffer solution.
  5. Divide the cell suspension into two fractions, each of 700 µL, in microcentrifuge tubes. Label the tubes as *dH_2_O* and *NaClO*.
  6. Add 150 µL of distilled water to the tube labeled *dH_2_O* and 150 µL of sodium hypochlorite [from a 5.5% (w/v) stock solution, commercially available household bleach] to the tube labeled *NaClO*. The final concentration of NaClO will be 1%. Gently mix to homogenize.
  7. Incubate at 37 °C for 30 min.
  8. After incubation, gently homogenize and measure the OD_600nm_ for each tube within 15 min after completion of incubation.
  9. The percentage of accumulated PHA is estimated using the formula described below in the Data analysis section (see examples in Figure 1).
    **Figure 1.**
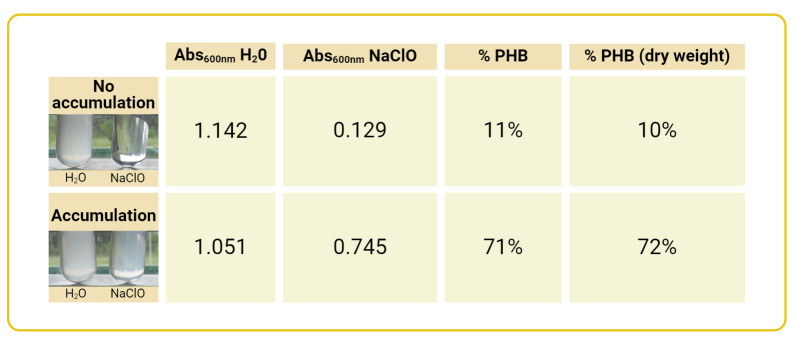
Estimation of polyhydroxybutyrate (PHB) content by Na-hypochlorite and dry weight. Values obtained with the described NaClO method are compared with the ones obtained after treating the cells with Na-hypochlorite for 30 min at 37 °C, followed by two washes with water and one wash with ethanol. % PHB (dry weight) content was then calculated as: [(mg/mL dry PHB)/(mg/mL dry cells)] × 100

## Data analysis

The following formula is used to estimate the percentage of accumulated PHB after Na-hypochlorite incubation:

$$\frac{{OD}_{600nm}\;of\;the\;hypochlorite\;treated\;sample\;(‘NaClO’)}{{OD}_{600nm}\;of\;the\;control\;sample\;(‘dH_{2}O’)}\times 100$$

## Validation of protocol

This protocol has been used and validated in the following research article:

Rodríguez et al. [3]. Characterization of polyhydroxybutyrate production from *Halomonas titanicae* KHS3 and manufacturing of electrosprayed nanoparticles. *Journal of Applied Polymer Science*. 141(6): e54928. (Supplementary information S1)

**Figure 2.**
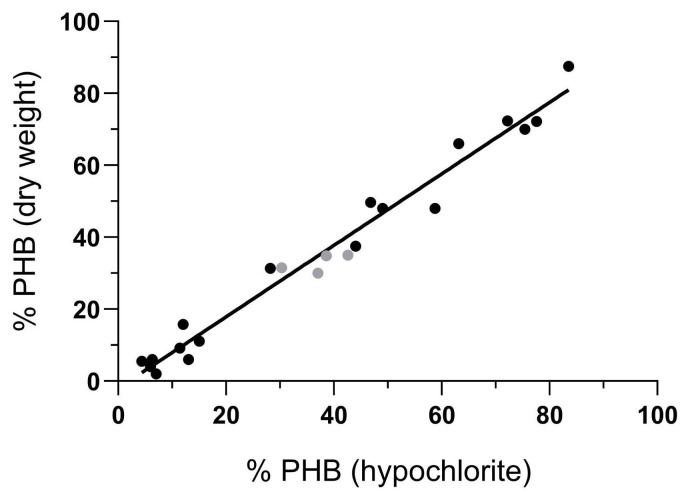
Graphical correlation between the percentage of PHB estimated with hypochlorite and other methods. The black circles indicate the correlation of PHB estimation vs. the percentage of PHB by dry weight; the gray circles indicate a correlation with the percentage of PHB obtained from quantification on HPLC. The R^2^ was 0.9733. Obtained from Rodríguez et al. [3].

The percentage of PHB estimated with hypochlorite was graphically correlated with the percentage using other methods (Figure 2). The values used for this analysis (Table 1) were obtained in multiple experiments in different conditions.

**Table 1.** Correlation between the content of PHB estimated with hypochlorite and dry weight. The values were obtained in multiple experiments in different conditions. *Obtained by HPLC quantification.

% PHA (hypochlorite)	% PHA (dry weight)
11.39	9.17
49	48
5.99	4.07
4.37	5.50
15	11.11
28.20	31.35
7	2
83.50	87.50
12	15.80
77.60	72.20
13	6
58.77	48
75.41	70
63.15	66
72.20	72.38
6.30	6.04
46.80	49.70
44	37.50
37	30*
42.60	35*
30.33	31.35*
38.60	34.80*

Dry cell weight was determined by the gravimetric method using 20 mL of culture and harvesting cells by centrifugation at 7,600× *g* for 10 min at 4 °C. Supernatants were discarded, and cell pellets were washed twice with distilled water. Pellets were dried at 105 °C up to constant weight.

Polymer extraction was performed as described previously by Williamson and Wilkinson [2] with slight modifications. This method is based on the resistance of PHA to sodium hypochlorite treatment. Alkaline sodium hypochlorite solution was added to the culture to a 1% w/v final concentration and incubated at 37 °C for 1 h. Then, the mixture was centrifuged at 9,700× *g* for 10 min at 4 °C, and the polymer pellet was collected and washed twice with distilled water and finally washed with ethanol 96%. The pellet was dried at 65 °C up to constant weight.

## General notes and troubleshooting

**General notes**

1. When using this protocol with new microorganisms, verify that the extracted compound is a PHB by nuclear magnetic resonance (NMR) or Fourier-transform infrared spectroscopy (FTIR). The correlation between this PHB estimation method and other PHB quantification methods should be verified for each different microorganism to be tested.
2. This protocol was evaluated on extreme halophilic haloarchaea, but no reliable results were obtained.

**Troubleshooting**

Problem 1: False positive.

Possible cause: Undiluted household bleach has a shelf life of six months to one year; beyond this period, it degrades and loses its oxidative activity.

Solution: Use fresh hypochlorite solution. Check that non-accumulating cultures are clarified after NaClO treatment.

Problem 2: Values of accumulation are much higher than expected.

Possible cause: If the optical density of the initial suspension is too high, it is possible that the concentration of NaClO or the incubation time were not enough for the complete dissolution of cellular content.

Solution: Be certain that the optical density of the initial cell suspension is between 1.7 and 2.

Problem 3: Too low OD_600nm_ values in the control sample (“dH_2_O”).

Possible cause: Sometimes, bacterial cells do not sediment properly after centrifugation, making it difficult to obtain an appropriate measurement of OD_600nm_ values for the control sample (“dH_2_O”). This could lead to OD_600nm_ values lower than 0.5 for this control sample, and this sample is excluded from the analysis.

Solution: Higher centrifugation forces could improve bacterial cell harvesting.
